# Predictors of Stroke Recurrence and Outcomes in Cerebral Small Vessel Disease: A Systematic Review of the Secondary Prevention of Small Subcortical Strokes (SPS3) Trial Findings

**DOI:** 10.7759/cureus.81393

**Published:** 2025-03-28

**Authors:** Saba Ahmed, Dushan Bosotov, Moshammet Manzia Noor, Mehak Gul, Fatima A Nassar, Sidra Anwar, Sundas Farhat, Muhammad Hassan Saleem, Arfan Irshad, Safdar Khan

**Affiliations:** 1 Anesthesia and Critical Care, Health Service Executive (HSE), Tralee, IRL; 2 Neurosurgery, American University of Antigua, New York, USA; 3 Neuroscience, Queen’s Hospital, London, GBR; 4 Internal Medicine, Jinnah Sindh Medical University, Karachi, PAK; 5 Neurology, Dubai Medical College for Girls, Dubai, ARE; 6 Internal Medicine, Shaikh Zayed Hospital, Lahore, PAK; 7 Internal Medicine, Fatima Jinnah Medical University, Lahore, PAK; 8 Medicine and Surgery, Shahida Islam Medical Complex, Lodhran, PAK; 9 College of Medicine, Caucasus's International University, Tbilisi, GEO; 10 Surgery, Services Hospital Lahore, Lahore, PAK

**Keywords:** cerebral small vessel disease, diabetes mellitus, dual antiplatelet therapy, high-sensitivity c-reactive protein, hypertension, inflammatory markers, lacunar stroke, secondary prevention of small subcortical strokes, stroke recurrence, vascular risk factors

## Abstract

Cerebral small vessel disease (CSVD) plays a significant role in the development of lacunar strokes and is closely associated with cognitive decline, gait disturbances, and vascular dementia. This systematic review explores the pathophysiology and management of CSVD, with a focus on its contribution to stroke recurrence and patient outcomes. Key findings indicate that structural cardiac abnormalities, inflammatory markers, and metabolic disorders are critical predictors of stroke risk in individuals with CSVD. Structural changes in the heart, such as altered left ventricular geometry, are linked to higher stroke recurrence rates, while elevated inflammatory markers, like high-sensitivity C-reactive protein, are associated with increased vascular events. Metabolic conditions, particularly diabetes, correlate with more severe vascular abnormalities and a heightened risk of recurrent strokes and mortality. Notably, some treatment strategies, such as dual antiplatelet therapy, may inadvertently increase mortality in specific patient groups, underscoring the importance of individualized therapeutic approaches. These insights emphasize the multifactorial nature of CSVD and highlight the need for comprehensive risk assessments and targeted management strategies to improve clinical outcomes.

## Introduction and background

Cerebral small vessel disease (CSVD) represents a spectrum of pathological processes affecting the small arteries, arterioles, venules, and capillaries of the brain [1]. It is a leading cause of lacunar strokes and contributes significantly to cognitive decline, gait disturbances, and vascular dementia [2]. The clinical manifestations of CSVD, particularly lacunar strokes, often present subtly but carry substantial long-term morbidity. Despite advancements in neuroimaging and diagnostic tools, CSVD remains underdiagnosed and undertreated, largely due to its complex pathophysiology and the variability in its clinical presentations [3]. The pathophysiological mechanisms underlying CSVD involve a combination of endothelial dysfunction, blood-brain barrier disruption, chronic hypoperfusion, and inflammatory processes, all contributing to the degeneration of small cerebral vessels [4].

The management of CSVD and its contribution to stroke requires a multifaceted approach, targeting both the modification of vascular risk factors and the prevention of recurrent strokes [5]. Current therapeutic strategies focus on controlling hypertension, diabetes, and hyperlipidemia, alongside the use of antiplatelet therapy. However, the efficacy of these interventions varies, and there is a pressing need to evaluate emerging therapeutic options and management protocols specifically tailored to CSVD [6]. This systematic review aims to synthesize the latest evidence on the pathophysiology and management of CSVD, emphasizing its role in stroke development and progression. By analyzing recent clinical trials and studies, this review seeks to provide a comprehensive understanding of the current landscape and identify potential gaps in research and practice.

To systematically address the research objectives, the PICO (Population, Intervention, Comparison, Outcome) framework guides the formulation of the review question and the selection of relevant studies [7]. The Population (P) includes adult patients diagnosed with CSVD, particularly those who have experienced lacunar strokes or other ischemic events attributed to CSVD. The Intervention (I) encompasses various therapeutic strategies aimed at managing CSVD and preventing stroke, including antihypertensive treatments, antiplatelet therapy, and lifestyle modifications. The Comparison (C) involves standard care practices or alternative therapeutic approaches, such as differing antihypertensive regimens, novel pharmacological agents, or non-pharmacological interventions. The Outcome (O) focuses on the incidence of stroke recurrence, cognitive function, quality of life (QOL), and other health-related outcomes associated with CSVD management. This PICO framework ensures a structured approach to identifying, evaluating, and synthesizing the evidence related to the pathophysiology and management of CSVD, ultimately contributing to more effective clinical practices and improved patient outcomes.

## Review

Materials and methods

Search Strategy

The search strategy for this systematic review adhered to the Preferred Reporting Items for Systematic Reviews and Meta-Analyses (PRISMA) guidelines [8] to ensure a transparent, comprehensive, and reproducible selection process. We conducted a systematic search across multiple electronic databases, including PubMed, MEDLINE, and Cochrane Library, focusing on studies related to CSVD and its contribution to stroke. Keywords such as “cerebral small vessel disease,” “lacunar stroke,” “stroke recurrence,” “quality of life post-stroke,” “infarct morphology,” and “vascular risk factors” were used in combination with Boolean operators to refine search results. Only randomized controlled trials (RCTs), cross-sectional analyses, and prospective biomarker studies published in peer-reviewed journals were included. Studies were screened based on titles and abstracts, followed by full-text reviews to ensure relevance and adherence to inclusion criteria. The selection process was documented using the PRISMA flow diagram, outlining the number of studies identified, screened, included, and excluded, along with justifications for exclusions.

Eligibility Criteria

The eligibility criteria for this systematic review were designed to ensure the inclusion of high-quality, relevant studies that provide comprehensive insights into CSVD and its contribution to stroke. We included RCTs, cross-sectional analyses, and prospective biomarker studies that specifically focused on lacunar strokes, vascular risk factors, infarct morphology, and stroke recurrence. Studies had to involve adult patients (aged 18 and above) diagnosed with CSVD or lacunar stroke, confirmed through neuroimaging techniques such as magnetic resonance imaging (MRI), diffusion-weighted imaging (DWI), or magnetic resonance angiography (MRA). Only peer-reviewed articles published in English within the last 15 years were considered to ensure the inclusion of recent, clinically relevant data. Additionally, studies needed to report on key outcomes such as stroke recurrence, QOL, mortality, or the predictive value of biomarkers in stroke prognosis.

Exclusion criteria included case reports, reviews, editorials, and studies focusing on stroke subtypes unrelated to CSVD, such as cardioembolic or large artery atherosclerotic strokes. Studies involving pediatric populations or animal models were also excluded, as were those lacking full-text availability or sufficient methodological rigor. Articles with incomplete data, unclear diagnostic criteria for CSVD, or non-standardized outcome measures were omitted to maintain the integrity of the review. This rigorous selection process ensured that only studies with robust methodologies and relevant findings were included, providing a solid foundation for analyzing the pathophysiology and management of CSVD in relation to stroke outcomes.

Data Extraction

Data extraction for this systematic review was conducted systematically and in accordance with PRISMA guidelines to ensure consistency and accuracy. A standardized data extraction form was used to collect key information from each included study, such as author names, publication year, study design, sample size, population characteristics, interventions, comparisons, and primary outcomes. Specific details on stroke recurrence rates, QOL assessments, infarct morphology, vascular risk factors, and biomarkers like high-sensitivity C-reactive protein (hsCRP) were also recorded. Two independent reviewers extracted the data to minimize bias, with discrepancies resolved through discussion or consultation with a third reviewer. Additionally, methodological details, such as randomization procedures and follow-up durations, were captured to facilitate quality assessment. The extracted data were organized in tables to allow for clear comparison across studies, supporting a comprehensive synthesis of findings related to the pathophysiology and management of CSVD and its role in stroke outcomes.

Data Analysis and Synthesis

Data analysis and synthesis for this systematic review were performed using a qualitative approach, focusing on identifying patterns, trends, and discrepancies across the included studies. Given the heterogeneity in study designs, populations, and outcome measures, such as QOL assessments, infarct morphology, and biomarkers like hsCRP, a meta-analysis was not feasible. Instead, a narrative synthesis was conducted to integrate findings from RCTs, cross-sectional analyses, and prospective biomarker studies. The results were compared and contrasted to highlight commonalities in the predictors of stroke recurrence, the impact of various interventions, and the role of comorbidities such as diabetes and hypertension. Special attention was given to unexpected outcomes, like the increased mortality observed with dual antiplatelet therapy, to explore potential methodological or contextual factors influencing these results. This comprehensive synthesis provided a cohesive understanding of the pathophysiology and management of CSVD in relation to stroke outcomes.

Results

Study Selection Process

**Figure 1:**
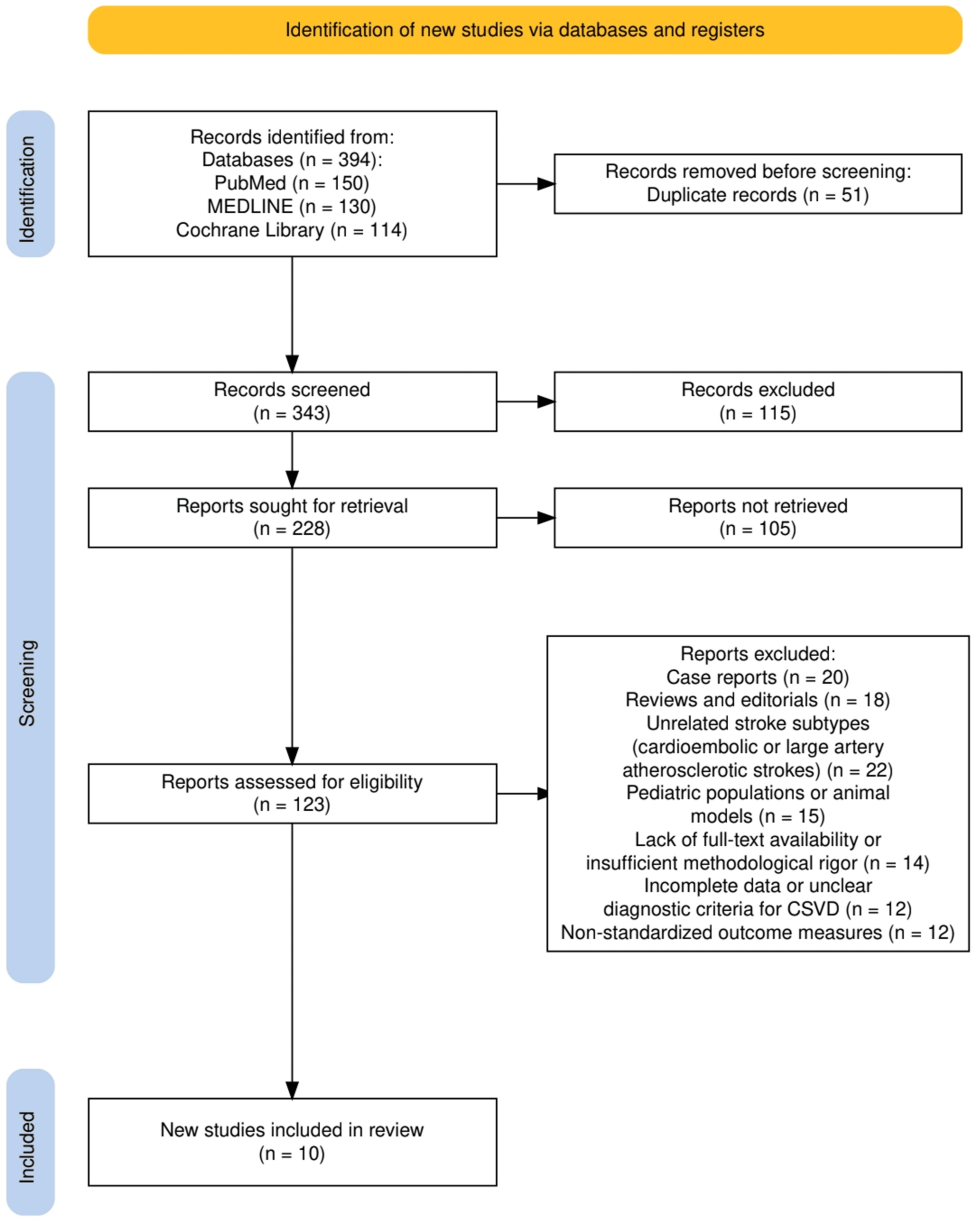
The PRISMA flowchart represents the study selection process. PRISMA: Preferred Reporting Items for Systematic Reviews and Meta-Analyses; CSVD: cerebral small vessel disease

The study selection process adhered to PRISMA guidelines and is illustrated in Figure 1. A total of 394 records were identified from three electronic databases: PubMed (n = 150), MEDLINE (n = 130), and the Cochrane Library (n = 114). After removing 51 duplicate records, 343 records were screened based on titles and abstracts. Of these, 115 records were excluded for not meeting the inclusion criteria. The remaining 228 reports were sought for retrieval, but 105 could not be retrieved. A total of 123 reports were assessed for eligibility through full-text review. Subsequently, 113 reports were excluded due to reasons such as case reports (n = 20), reviews and editorials (n = 18), unrelated stroke subtypes like cardioembolic or large artery atherosclerotic strokes (n = 22), studies involving pediatric populations or animal models (n = 15), lack of full-text availability or insufficient methodological rigor (n = 14), incomplete data or unclear diagnostic criteria for CSVD (n = 12), and non-standardized outcome measures (n = 12). Ultimately, 10 studies met all eligibility criteria and were included in the final systematic review.

Characteristics of the Selected Studies

The characteristics of the selected studies are summarized in Table 1. The studies predominantly consist of RCTs from the Secondary Prevention of Small Subcortical Strokes (SPS3) trial, alongside cross-sectional analyses and prospective biomarker studies. The populations studied included lacunar stroke patients, with sample sizes ranging from 644 to 3,020 participants, and demographic variations such as age, sex, and comorbid conditions like diabetes. Interventions varied across studies, including QOL assessments, MRI-based infarct morphology classification, dual antiplatelet therapy trials, and biomarker measurements such as hsCRP. Comparisons were drawn between different risk factors, such as diabetic versus non-diabetic patients, patients with or without vertebrobasilar ectasia (VBE), and variations in infarct morphology. Key findings indicated that factors like abnormal left ventricular geometry (LVG), high hsCRP levels, and dual antiplatelet therapy were linked to increased risks of stroke recurrence and mortality. Furthermore, infarct shapes and volumes influenced short-term functional outcomes, while vascular risk factors like hypertension and diabetes were consistently associated with poorer prognoses. These diverse findings underscore the multifaceted role of CSVD in stroke outcomes.

**Table 1:** The characteristics of the included studies. RCT: randomized controlled trial; SPS3: Secondary Prevention of Small Subcortical Strokes; QOL: quality of life; SSQOL: Stroke Specific Quality of Life Score; MRI: magnetic resonance imaging; LV: left ventricular; LVG: left ventricular geometry; HR: hazard ratio; SBP: systolic blood pressure; mm Hg: millimeters of mercury; DWI: diffusion-weighted imaging; BP: blood pressure; MRA: magnetic resonance angiography; CTA: computed tomography angiography; VBE: vertebrobasilar ectasia; hsCRP: high-sensitivity C-reactive protein; limits: levels of inflammatory markers in the treatment of stroke; BMI: body mass index; TIA: transient ischemic attack

Authors	Study Design	Population	Intervention	Comparison	Key Findings
Dhamoon MS et al. (2014) [9]	RCT (SPS3)	2870 lacunar stroke patients (mean age 63.4 years, 63% male)	Annual QOL assessments using SSQOL	None	QOL increased 0.6% annually post-stroke. Age, prior stroke, and college education were associated with decline in QOL over time. Multiple strokes led to long-term QOL decline even without recurrent events.
Field TS et al. (2015) [10]	RCT (SPS3)	1961 lacunar stroke patients with MRI-confirmed diagnosis	Transthoracic echocardiography assessment of LV geometry	Standard prognosis assessments	Abnormal LV geometry is present in 77% of patients, linked to higher risk of stroke recurrence (HR 1.5). No association with mortality. LVG correlated with small-vessel disease manifestations and chronic hypertension.
White CL et al. (2013) [11]	Cross-sectional analysis (SPS3)	3,020 lacunar stroke patients	Baseline blood pressure assessment and antihypertensive medication use	None	53% had SBP ≥ 140 mm Hg, and 18% had SBP ≥ 160 mm Hg despite treatment. Higher SBP is linked to more extensive white matter disease. Regional and racial differences influenced SBP control; Black participants were more likely to have uncontrolled hypertension.
Wilson LK et al. (2016) [12]	RCT (SPS3)	644 patients with MRI-confirmed pontine infarcts	Morphological classification of infarcts using MRI	Comparison of infarct types (small deep vs. paramedian)	49% had small deep infarcts (lacunar), 40% paramedian wedge infarcts. Smoking was independently associated with small deep infarcts. No significant differences in stroke recurrence or mortality based on infarct morphology. Treatment strategies had no differential effect based on infarct type.
Sharma M et al. (2014) [13]	RCT (SPS3)	3,020 lacunar stroke patients (mean age 63 years)	Dual antiplatelet therapy (clopidogrel + aspirin) vs. aspirin alone	Single antiplatelet therapy (aspirin)	Dual antiplatelet therapy was linked to increased all-cause mortality, especially in patients with ischemic heart disease and those who were normotensive/prehypertensive. Nonfatal major hemorrhage significantly raised mortality risk (HR 4.5). Predictors of mortality included age, diabetes, hypertension, renal function, low hemoglobin, and lower BMI.
Asdaghi N et al. (2014) [14]	RCT (SPS3)	1,679 lacunar stroke patients with DWI-confirmed infarcts	Classification of infarct shape and measurement of volume using MRI	Comparison of infarct shapes and volumes	Infarct shapes included ovoid/spheroid (63%), slab (12%), stick (7%), and multicomponent (17%). Larger infarct volumes correlated with worse short-term functional outcomes but did not predict recurrent stroke. Vascular risk factors were similar across infarct types, except for higher diabetes prevalence in ovoid/spheroid and stick shapes, and higher BP in multicomponent infarcts.
Nakajima M et al. (2015) [15]	RCT (SPS3)	2,621 lacunar stroke patients assessed via MRA/CTA	Measurement of vertebrobasilar artery diameters to identify VBE	Patients with vs. without VBE	VBE was present in 7.6% of patients. VBE was predictive of increased mortality (HR 1.7) but not of recurrent stroke or major hemorrhage. VBE was associated with higher diastolic BP and inversely related to systolic BP. Risk factors included older age, male sex, White ethnicity, and hypertension.
Elkind MS et al. (2014) [16]	Prospective biomarker study (LIMITS nested within SPS3 RCT)	1,244 lacunar stroke patients (mean age 63.3 years)	Measurement of hsCRP levels	Comparison across hsCRP quartiles	Patients in the top hsCRP quartile (>4.86 mg/L) had a higher risk of recurrent ischemic stroke (adjusted HR 2.32) and major vascular events (adjusted HR 2.04). hsCRP was a strong predictor of stroke recurrence and vascular events but did not influence the response to antiplatelet therapy.
Palacio S et al. (2014) [17]	RCT (SPS3)	3,020 lacunar stroke patients, 37% with diabetes mellitus	Comparison of diabetic vs. non-diabetic patients regarding risk factors, infarct location, and prognosis	Patients without diabetes mellitus	Diabetic patients had higher rates of intracranial stenosis, posterior circulation infarcts, and white matter abnormalities. Diabetes doubled the risk of recurrent stroke (HR 1.8), myocardial infarction (HR 1.7), and death (HR 2.1). Diabetics showed a distinctive profile with worse outcomes compared to non-diabetics.
Hart RG et al. (2014) [18]	RCT (SPS3)	3,020 lacunar stroke patients followed for 3.7 years	Aggressive BP control and dual antiplatelet therapy vs. standard care	Comparison based on stroke recurrence risk status	Prior symptomatic lacunar stroke/TIA (HR 2.2), diabetes (HR 2.0), Black race (HR 1.7), and male sex (HR 1.5) predicted recurrent stroke. High-risk patients had a recurrence rate of 4.3%/yr, while low-risk patients had 1.3%/yr. No significant interaction between risk factors and treatment effects.

Quality Assessment

The quality assessment of the included studies is detailed in Table 2, utilizing standardized tools such as the Cochrane Risk of Bias Tool [19], the Appraisal tool for Cross-Sectional Studies (AXIS tool) [20], and Quality Assessment of Diagnostic Accuracy Studies (QUADAS-2) [21]. The majority of the RCTs, including those by Dhamoon MS et al. [9], Field TS et al. [10], and Wilson LK et al. [12], were assessed as having a low risk of bias due to their robust methodologies, high inter-rater reliability, and well-defined measurement criteria. Studies like Asdaghi N et al. [14] and Nakajima M et al. [15] also demonstrated low risk, attributed to comprehensive imaging analyses and precise diagnostic measurements. However, Sharma M et al. [13] showed a moderate risk of bias due to unexpected mortality interactions related to dual antiplatelet therapy. The cross-sectional study by White CL et al. [11] exhibited a moderate risk of bias, primarily due to inherent limitations in its study design, which affected causality inference. The biomarker study by Elkind MSV et al. [16] was evaluated using QUADAS-2 and demonstrated a low risk of bias due to strong validation of hsCRP as a predictor. Overall, the studies included in this review were of high methodological quality, ensuring reliable and valid conclusions.

**Table 2:** A summary of the quality assessment of the included studies. AXIS tool: Appraisal tool for Cross-Sectional Studies; QUADAS-2: Quality Assessment of Diagnostic Accuracy Studies

Authors	Quality Assessment Tool	Quality Assessment
Dhamoon MS et al. [9]	Cochrane Risk of Bias Tool [19]	Low risk of bias, robust methodology
Field TS et al. [10]	Cochrane Risk of Bias Tool [19]	Low risk of bias, high external validity
White CL et al. [11]	AXIS Tool [20]	Moderate risk of bias due to cross-sectional design
Wilson LK et al. [12]	Cochrane Risk of Bias Tool [19]	Low risk of bias, high inter-rater reliability
Sharma M et al. [13]	Cochrane Risk of Bias Tool [19]	Moderate risk of bias due to unexpected mortality interaction
Asdaghi N et al. [14]	Cochrane Risk of Bias Tool [19]	Low risk of bias, comprehensive imaging analysis
Nakajima M et al. [15]	Cochrane Risk of Bias Tool [19]	Low risk of bias, well-defined measurement criteria
Elkind MS et al. [16]	QUADAS-2 [21]	Low risk of bias, strong biomarker validation
Palacio S et al. [17]	Cochrane Risk of Bias Tool [19]	Low risk of bias, comprehensive subgroup analysis
Hart RG et al. [18]	Cochrane Risk of Bias Tool [19]	Low risk of bias, consistent findings across risk groups

Discussion

The systematic review of studies from the SPS3 trial presents several key findings that illuminate the complex interplay between CSVD and stroke outcomes. Dhamoon MS et al. [9] found a modest annual improvement in QOL post-lacunar stroke, though factors like age, prior stroke, and higher education were associated with a decline over time. Field TS et al. [10] reported that 77% of lacunar stroke patients exhibited abnormal LVG, correlating with increased stroke recurrence risk but not mortality. White CL et al. [11] highlighted the prevalence of uncontrolled hypertension in 53% of lacunar stroke patients, with significant racial and regional disparities in blood pressure control. Wilson LK et al. [12] classified a morphological classification of pontine infarcts and found no significant differences in recurrence or mortality based on infarct type, although smoking was a notable risk factor. Additionally, Sharma M et al. [13] revealed that dual antiplatelet therapy increased all-cause mortality, particularly in patients with ischemic heart disease and normotension, while nonfatal major hemorrhage significantly raised mortality risks.

Interpreting these findings within the broader context of stroke research reveals both consistent patterns and novel insights. The association of LVG with stroke recurrence, as shown by Field TS et al. [10], aligns with existing knowledge of the cardiovascular contributions to cerebrovascular events, reinforcing the link between chronic hypertension and CSVD. Conversely, the findings of Wilson LK et al. [12] challenge the assumption that infarct morphology significantly influences stroke outcomes, suggesting that other factors like systemic vascular health may play more critical roles. The study by Elkind MS et al. [16] on hsCRP as a predictor of recurrent stroke adds to the growing evidence of inflammation's role in stroke pathology, while Palacio S et al. [17] underscored the heightened risks faced by diabetic patients, further corroborating diabetes as a key modifiable risk factor in stroke prevention strategies. These studies collectively contribute to a nuanced understanding of CSVD and its management, offering valuable directions for future research and clinical practice.

When comparing the findings of the SPS3 trial studies with existing literature, several consistencies and discrepancies emerge. The observation by Field TS et al. [10] that abnormal LVG correlates with increased stroke recurrence aligns with prior studies, highlighting the cardiovascular contributions to cerebrovascular events and reinforcing the role of chronic hypertension as a key factor in CSVD progression. Similarly, Elkind MS et al. [16] confirmed that elevated hsCRP levels predict recurrent ischemic strokes, which is consistent with the established link between systemic inflammation and vascular events. However, the findings by Wilson LK et al. [12], which indicated no significant differences in stroke recurrence or mortality based on infarct morphology, contrast with earlier studies that suggested infarct size and shape significantly influence outcomes. This discrepancy may be attributed to differences in study design, sample size, or the specific characteristics of the patient populations under investigation.

Unexpected results were notably observed in Sharma M et al. [13], where dual antiplatelet therapy (clopidogrel plus aspirin) was associated with increased all-cause mortality, particularly in patients with ischemic heart disease and those who were normotensive or prehypertensive. This finding contradicts previous research suggesting dual antiplatelet therapy offers superior protection against recurrent strokes [22]. One possible explanation could be an increased risk of nonfatal major hemorrhages, as highlighted by the study, which may have outweighed the benefits of stroke prevention [23]. Methodological factors, such as the inclusion criteria for patients and the specific blood pressure targets, could also have influenced these outcomes. Additionally, regional variations in treatment protocols and genetic predispositions in different populations might have contributed to the unexpected mortality rates. These results underscore the importance of individualized treatment strategies in managing CSVD and stroke prevention.

The findings from the SPS3 trial studies have significant theoretical and clinical implications for the management of CSVD and stroke prevention. Clinically, the association between abnormal LVG and increased stroke recurrence, as reported by Field TS et al. [10], highlights the need for routine cardiovascular assessments in stroke patients to identify those at higher risk of recurrence. The predictive value of hsCRP levels for recurrent ischemic strokes, as demonstrated by Elkind MS et al. [16], suggests that inflammatory markers could be integrated into standard stroke risk assessment protocols, potentially guiding personalized treatment strategies. The unexpected increase in mortality with dual antiplatelet therapy observed by Sharma M et al. [13] underscores the necessity for careful patient selection and risk stratification when considering aggressive antithrombotic regimens, particularly in those with ischemic heart disease or normotension. Theoretically, these studies advance the understanding of CSVD’s multifactorial nature [24], emphasizing the interplay between vascular, inflammatory, and structural cardiac factors. Collectively, these findings could inform clinical guidelines, encouraging a more individualized approach to stroke prevention and prompting further research into targeted therapies for high-risk populations [25].

The SPS3 trial studies exhibit several strengths that enhance the reliability and applicability of their findings. Notably, the large, diverse sample sizes, ranging from over 1,200 to more than 3,000 participants, provide robust statistical power and improve the generalizability of the results across different populations. The use of advanced neuroimaging techniques, such as MRI and DWI, allowed for precise classification of infarct morphology and volume, while the incorporation of biomarkers like hsCRP offered novel insights into the inflammatory pathways involved in stroke recurrence. Additionally, the multicenter, RCT design ensured a high level of methodological rigor, reducing potential biases. However, some limitations must be acknowledged. The reliance on specific patient populations, particularly those with lacunar strokes, may limit the generalizability of findings to other stroke subtypes. Moreover, certain unexpected outcomes, such as increased mortality with dual antiplatelet therapy, might have been influenced by unmeasured confounding variables or variations in treatment adherence across centers. These limitations highlight the need for cautious interpretation of the results and suggest that further studies are necessary to confirm these findings in broader clinical contexts.

Future research should focus on addressing the gaps identified in the SPS3 trial studies, particularly the need for more individualized treatment strategies for patients with CSVD. Given the unexpected increase in mortality associated with dual antiplatelet therapy, further studies should explore the underlying mechanisms of this adverse outcome, potentially through stratified analyses based on genetic factors, comorbidities, or regional differences in healthcare practices. Additionally, longitudinal studies examining the long-term effects of inflammatory markers like hsCRP on stroke recurrence could offer deeper insights into the role of systemic inflammation in CSVD progression [26,27]. Methodologically, future trials could benefit from incorporating advanced imaging techniques to monitor subtle changes in cerebral microvasculature over time and utilizing wearable technology for continuous blood pressure monitoring to assess real-time vascular health. Exploring the efficacy of emerging therapies, such as targeted anti-inflammatory agents or personalized antihypertensive regimens, would also help refine current management protocols and improve outcomes for high-risk populations.

## Conclusions

In conclusion, the findings from the SPS3 trial studies provide critical insights into the complex interplay of vascular, inflammatory, and structural factors contributing to stroke recurrence in patients with CSVD. The identification of key predictors such as abnormal LVG, elevated hsCRP levels, and diabetes underscores the need for comprehensive, individualized risk assessment in clinical practice. The unexpected risks associated with dual antiplatelet therapy highlight the importance of cautious therapeutic decision-making, particularly in patients with specific comorbidities. Collectively, these studies advance our understanding of CSVD's multifaceted nature and pave the way for more targeted, evidence-based interventions aimed at reducing stroke recurrence and improving long-term patient outcomes.

## References

[1] Gao Y, Li D, Lin J (2022). Cerebral small vessel disease: pathological mechanisms and potential therapeutic targets. Front Aging Neurosci.

[2] Cai Z, Wang C, He W, Tu H, Tang Z, Xiao M, Yan LJ (2015). Cerebral small vessel disease and Alzheimer's disease. Clin Interv Aging.

[3] Gore M, Bansal K, Khan Suheb MZ, Lui F, Asuncion RMD (2024). Lacunar stroke. StatPearls [Internet].

[4] Rajeev V, Fann DY, Dinh QN (2022). Pathophysiology of blood brain barrier dysfunction during chronic cerebral hypoperfusion in vascular cognitive impairment. Theranostics.

[5] Ren B, Tan L, Song Y, Li D, Xue B, Lai X, Gao Y (2022). Cerebral small vessel disease: neuroimaging features, biochemical markers, influencing factors, pathological mechanism and treatment. Front Neurol.

[6] Borghi C, Fogacci F, Agnoletti D, Cicero AF (2022). Hypertension and dyslipidemia combined therapeutic approaches. High Blood Press Cardiovasc Prev.

[7] Brown D (2020). A review of the PubMed PICO tool: using evidence-based practice in health education. Health Promot Pract.

[8] Page MJ, McKenzie JE, Bossuyt PM (2021). The PRISMA 2020 statement: an updated guideline for reporting systematic reviews. BMJ.

[9] Dhamoon MS, McClure LA, White CL, Lau H, Benavente O, Elkind MS (2014). Quality of life after lacunar stroke: the Secondary Prevention of Small Subcortical Strokes study. J Stroke Cerebrovasc Dis.

[10] Field TS, Pearce LA, Asinger RW, Smyth NG, De SK, Hart RG, Benavente OR (2015). Left ventricular geometry on transthoracic echocardiogram and prognosis after lacunar stroke: the SPS3 trial. J Stroke Cerebrovasc Dis.

[11] White CL, Pergola PE, Szychowski JM (2013). Blood pressure after recent stroke: baseline findings from the secondary prevention of small subcortical strokes trial. Am J Hypertens.

[12] Wilson LK, Pearce LA, Arauz A (2016). Morphological classification of penetrating artery pontine infarcts and association with risk factors and prognosis: the SPS3 trial. Int J Stroke.

[13] Sharma M, Pearce LA, Benavente OR (2014). Predictors of mortality in patients with lacunar stroke in the secondary prevention of small subcortical strokes trial. Stroke.

[14] Asdaghi N, Pearce LA, Nakajima M (2014). Clinical correlates of infarct shape and volume in lacunar strokes: the Secondary Prevention of Small Subcortical Strokes trial. Stroke.

[15] Nakajima M, Pearce LA, Ohara N (2015). Vertebrobasilar ectasia in patients with lacunar stroke: the Secondary Prevention of Small Subcortical Strokes trial. J Stroke Cerebrovasc Dis.

[16] Elkind MS, Luna JM, McClure LA (2014). C-reactive protein as a prognostic marker after lacunar stroke: levels of inflammatory markers in the treatment of stroke study. Stroke.

[17] Palacio S, McClure LA, Benavente OR, Bazan C 3rd, Pergola P, Hart RG (2014). Lacunar strokes in patients with diabetes mellitus: risk factors, infarct location, and prognosis: the secondary prevention of small subcortical strokes study. Stroke.

[18] Hart RG, Pearce LA, Bakheet MF (2014). Predictors of stroke recurrence in patients with recent lacunar stroke and response to interventions according to risk status: Secondary Prevention of Small Subcortical Strokes trial. J Stroke Cerebrovasc Dis.

[19] Higgins JP, Altman DG, Gøtzsche PC (2011). The Cochrane Collaboration's tool for assessing risk of bias in randomised trials. BMJ.

[20] Downes MJ, Brennan ML, Williams HC, Dean RS (2016). Development of a critical appraisal tool to assess the quality of cross-sectional studies (AXIS). BMJ Open.

[21] Whiting PF, Rutjes AW, Westwood ME (2011). QUADAS-2: a revised tool for the quality assessment of diagnostic accuracy studies. Ann Intern Med.

[22] Ge F, Lin H, Liu Y, Li M, Guo R, Ruan Z, Chang T (2016). Dual antiplatelet therapy after stroke or transient ischaemic attack - how long to treat? The duration of aspirin plus clopidogrel in stroke or transient ischaemic attack: a systematic review and meta-analysis. Eur J Neurol.

[23] Bushnell CD, Colón-Emeric CS (2009). Secondary stroke prevention strategies for the oldest patients: possibilities and challenges. Drugs Aging.

[24] Gurol ME, Sacco RL, McCullough LD (2020). Multiple faces of cerebral small vessel diseases. Stroke.

[25] Bushnell C, Kernan WN, Sharrief AZ (2024). 2024 guideline for the primary prevention of stroke: a guideline from the American Heart Association/American Stroke Association. Stroke.

[26] Chen L, Wang M, Yang C, Wang Y, Hou B (2023). The role of high-sensitivity C-reactive protein serum levels in the prognosis for patients with stroke: a meta-analysis. Front Neurol.

[27] Wang Y, Li J, Pan Y, Wang M, Meng X, Wang Y (2022). Association between high-sensitivity C-reactive protein and prognosis in different periods after ischemic stroke or transient ischemic attack. J Am Heart Assoc.

